# Povidone-Iodine Induced Allergic Contact Dermatitis in a 10-Year-Old Girl: A Case Report

**DOI:** 10.7759/cureus.65121

**Published:** 2024-07-22

**Authors:** Bhavesh Kanabar, Kiran G Piparva, Rajvi B Kanabar

**Affiliations:** 1 Department of Preventive and Social Medicine, Pandit Deendayal Upadhyay Government Medical College, Rajkot, Rajkot, IND; 2 Department of Pharmacology, All India Institute of Medical Sciences, Rajkot, Rajkot, IND; 3 Department of Family Medicine, Rajkot Municipal Corporation, Rajkot, IND

**Keywords:** adr, povidone-iodine, pvp-i, dermatitis, antiseptics

## Abstract

We present a case of iodine-induced allergic contact dermatitis in a 10-year-old child. The child had a superficial wound on the left knee from an injury and was treated with daily applications of povidone-iodine (PVP-I) ointment for three to four days. The child subsequently developed a worsening skin lesion that increased from an initial 2 cm to 10 cm, spreading over the upper part of the leg, accompanied by pain and scanty discharge. Referred to the dermatology department, the dermatologist diagnosed iodine-induced allergic contact dermatitis based on the clinical presentation and the absence of other oral or topical medications, as well as no history of allergy to any substances or medications. Discontinuation of the suspected PVP-I ointment led to complete healing within 10 days with the use of only an emollient. This case underscores the importance of recognizing iodine allergy as a potential complication when used in wound care, particularly in pediatric patients.

## Introduction

Povidone-iodine (PVP-I) is an iodine-containing antiseptic and disinfectant. This antiseptic is widely popular for hand washing and wound care in hospital settings as well as at home. PVP-I is a commercially available formulation that contains polyvinylpyrrolidone. Shelanski HA and Shelanski MV first introduced PVP-I in 1956 [1]. PVP-I is a water-soluble formed by the combination of molecular iodine and polyvinylpyrrolidone. The 10% polyvinylpyrrolidone-iodine solution contains 90% water, 8.5% polyvinylpyrrolidone, and 1% each of available iodine and iodide [2]. PVP-I has broad-spectrum antimicrobial properties against bacteria, including anaerobic and sporulated organisms, fungi, protozoa, viruses, and certain bacterial spores [3].

When utilized appropriately, PVP-I is generally considered safe as an antiseptic, with an exceptionally low occurrence of allergy and contact dermatitis among normal subjects. Out of 5,000 applications, only two allergic reactions have been documented [4]. However, it has the potential to induce allergic contact dermatitis, which can lead to severe skin reactions [5]. Allergic contact dermatitis resulting from the application of PVP-I for wound care in paediatric cases is infrequently reported. We are presenting a similar case where allergic contact dermatitis developed after the application of PVP-I ointment in wound care in a paediatric case.

## Case presentation

**Figure 1:**
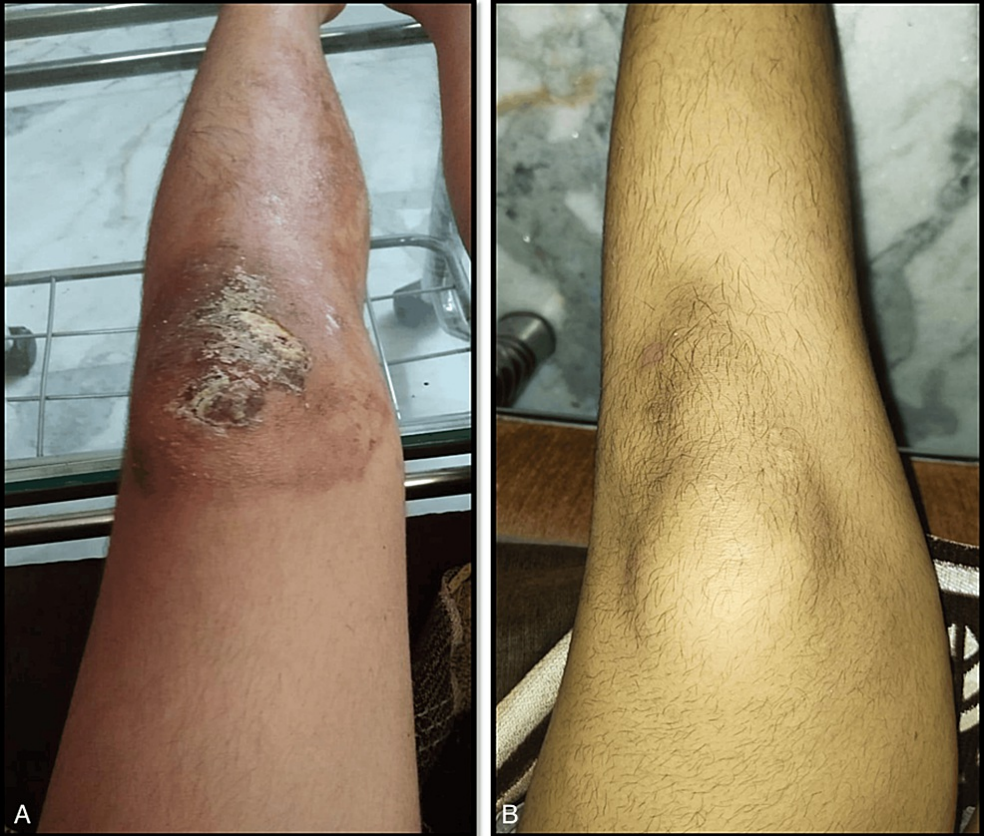
(A) Povidone-iodine-induced allergic dermatitis on the left knee; (B) Wound healed 10 days after discontinuation of povidone-iodine ointment. (A) The image was of a wound measuring 10 cm in length and 4 cm in width that spread from the knee to the upper part of the lower leg, after daily application of povidone-iodine ointment for three to four days. The wound showed yellowish exudates and had irregular margins with areas of undermining, indicating deeper tissue involvement. (B) After 10 days of discontinuing povidone-iodine ointment, the wound completely healed with the use of emollient only.

A 10-year-old girl child presented with a superficial wound on the left knee after an injury. The lesion was initially 0.5 cm in diameter and was treated with PVP-I 5% weight/weight (w/w) ointment application two to three times a day. After five days of PVP-I ointment application, however, instead of improving, the lesion increased in size to approximately 10 cm in length and 4 cm in width at its widest point. It spread to the upper part of the lower leg and became itchy and tender. The wound appeared yellowish exudates with irregular margins and undermined in some areas, suggesting the involvement of deeper tissues (Figure 1).

The child was referred to the dermatology department. There was no history of allergies to any substances or medications. There was no history of oral intake or topical application of any medications simultaneously. The dermatologist suspected allergic contact dermatitis induced by the PVP-I ointment based on clinical examination and history of exposure. The suspected PVP-I was stopped, and the lesion was managed with emollients. The lesion completely healed within 10 days.

According to WHO [6], the assessment of causality for allergic contact dermatitis induced by PVP-I was categorized as "probable". According to the Hartwig and Siegel criteria, the severity of adverse drug reaction (ADR) is classified as "Level 3" [7].

## Discussion

PVP-I can be applied prophylactically during wound cleaning and therapeutically as a leave-on treatment for both contaminated chronic and acute wounds [8]. Contamination with pathogenic microbes can lead to infection and sepsis, which can interfere with the healing process [9].

The role of iodine in wound care is primarily as an antimicrobial agent. The concentration of free iodine determines the germicidal action of PVP-I [10]. The absorption of iodine is affected by factors such as the concentration of the solution used, the frequency of applications, and the route of administration, specifically when applied topically on the skin [11]. However, the condition of the skin barrier will determine transdermal iodine absorption. The absorption will be increased if the skin barrier is broken as in wounds and also dependent on skin age and surface area of application [12]. Knolle and colleagues showed that absorption occurs through intact adult skin following five sessions of five-minute scrubbing with a polyvinylpyrrolidone-iodine scrub preparation [11]. Systemically absorbed iodine binds to serum albumin in the bloodstream and is subsequently eliminated by the kidneys [13].

Polyvinylpyrrolidone, the hydrophilic polymer that acts as a carrier in PVP-I, does not have any intrinsic antibacterial activity. Clinical administration of polyvinylpyrrolidone-iodine through any route may lead to systemic iodine absorption. While no cutaneous or mucosal absorption of polyvinylpyrrolidone has been observed in healthy laboratory animals, research on inflamed membranes is currently unavailable [3].

Due to the low concentration of free iodine in PVP-I, skin irritation is typically less pronounced during short-term contact [14]. Many researchers have reported instances of moderate to severe irritant contact dermatitis resulting from the application of PVP-I for preoperative painting and draping [15]. The equilibrium between povidone-bound iodine and free iodine in PVP-I formulations is pivotal for maximizing antimicrobial efficacy while minimizing safety concerns. By carefully adjusting the formulation, concentration, and temperature, PVP-I provides a stable and tolerable antiseptic for wound healing [16]. Preclinical and clinical research is actively exploring various formulations and concentrations. Nevertheless, despite these advancements, some cases of adverse events remain unanswered [10].

The lower concentration, shorter contact time, and intact skin barrier typically reduce the likelihood of systemic absorption of free iodine, thereby minimizing the risk of allergy. However, in the case of ointment formulation, prolonged retention and continuous absorption from the wound area, including raw and subcutaneous tissues, may facilitate iodine absorption. This can sensitize surrounding tissues and exacerbate dermatitis progression. Several cases in the literature have reported contact dermatitis from PVP-I solution used for painting and draping on large intact skin areas. This case presents contact dermatitis in pediatric patients when used for wound care in smaller sizes, highlighting its potential to induce allergic reactions.

## Conclusions

PVP-I is an antimicrobial agent known for its broad spectrum of activity. It is widely used as a potent antiseptic in treating wounds, skin conditions, and vaginal infections for preventing infections. However, its capacity to permeate the skin and subcutaneous tissues may lead to allergic contact dermatitis, especially in pediatrics. Healthcare providers should carefully balance its therapeutic benefits with potential risks, including allergic reactions when employing it for pediatric wound management. Understanding these considerations is crucial for optimizing patient safety and treatment outcomes.
